# Computed tomography-derived body composition and early changes predict outcomes of first-line chemo-immunotherapy in recurrent/metastatic nasopharyngeal carcinoma

**DOI:** 10.1093/oncolo/oyag355

**Published:** 2026-09-03

**Authors:** Xia Gan, Zhengzheng Yu, Yaning Tang, Lin Zhang, Wenji Xie, Xiaowen Sun, Yue Li, Jianing Fang, Yating Liu, Zhongjie Liu, Can Zhou, Zhuolong Xie, Wen Yao, Shuyu Ouyang, Jing Zhang, Jingyu Wang, Hua Li, Pan Chen, Hui Wang, Yanxian Li, Huai Liu

**Affiliations:** Department of Radiation Oncology, Hunan Cancer Hospital and the Affiliated Cancer Hospital of Xiangya School of Medicine, Central South University, Changsha, 410013, China; Hunan Key Laboratory of Translational Radiation Oncology, Hunan Cancer Hospital and The Affiliated Cancer Hospital of Xiangya School of Medicine, Central South University, Changsha, 410013, China; Department of Radiation Oncology, Hunan Cancer Hospital and the Affiliated Cancer Hospital of Xiangya School of Medicine, Central South University, Changsha, 410013, China; Hunan Key Laboratory of Translational Radiation Oncology, Hunan Cancer Hospital and The Affiliated Cancer Hospital of Xiangya School of Medicine, Central South University, Changsha, 410013, China; Department of Radiation Oncology, Hunan Cancer Hospital and the Affiliated Cancer Hospital of Xiangya School of Medicine, Central South University, Changsha, 410013, China; Hunan Key Laboratory of Translational Radiation Oncology, Hunan Cancer Hospital and The Affiliated Cancer Hospital of Xiangya School of Medicine, Central South University, Changsha, 410013, China; Department of Radiation Oncology, Hunan Cancer Hospital and the Affiliated Cancer Hospital of Xiangya School of Medicine, Central South University, Changsha, 410013, China; Hunan Key Laboratory of Translational Radiation Oncology, Hunan Cancer Hospital and The Affiliated Cancer Hospital of Xiangya School of Medicine, Central South University, Changsha, 410013, China; Department of Radiation Oncology, Hunan Cancer Hospital and the Affiliated Cancer Hospital of Xiangya School of Medicine, Central South University, Changsha, 410013, China; Hunan Key Laboratory of Translational Radiation Oncology, Hunan Cancer Hospital and The Affiliated Cancer Hospital of Xiangya School of Medicine, Central South University, Changsha, 410013, China; Department of Radiation Oncology, Hunan Cancer Hospital and the Affiliated Cancer Hospital of Xiangya School of Medicine, Central South University, Changsha, 410013, China; Hunan Key Laboratory of Translational Radiation Oncology, Hunan Cancer Hospital and The Affiliated Cancer Hospital of Xiangya School of Medicine, Central South University, Changsha, 410013, China; Department of Radiation Oncology, Hunan Cancer Hospital and the Affiliated Cancer Hospital of Xiangya School of Medicine, Central South University, Changsha, 410013, China; Hunan Key Laboratory of Translational Radiation Oncology, Hunan Cancer Hospital and The Affiliated Cancer Hospital of Xiangya School of Medicine, Central South University, Changsha, 410013, China; Department of Radiation Oncology, Hunan Cancer Hospital and the Affiliated Cancer Hospital of Xiangya School of Medicine, Central South University, Changsha, 410013, China; Hunan Key Laboratory of Translational Radiation Oncology, Hunan Cancer Hospital and The Affiliated Cancer Hospital of Xiangya School of Medicine, Central South University, Changsha, 410013, China; Department of Radiation Oncology, Hunan Cancer Hospital and the Affiliated Cancer Hospital of Xiangya School of Medicine, Central South University, Changsha, 410013, China; Hunan Key Laboratory of Translational Radiation Oncology, Hunan Cancer Hospital and The Affiliated Cancer Hospital of Xiangya School of Medicine, Central South University, Changsha, 410013, China; Department of Radiation Oncology, Hunan Cancer Hospital and the Affiliated Cancer Hospital of Xiangya School of Medicine, Central South University, Changsha, 410013, China; Hunan Key Laboratory of Translational Radiation Oncology, Hunan Cancer Hospital and The Affiliated Cancer Hospital of Xiangya School of Medicine, Central South University, Changsha, 410013, China; Department of Radiation Oncology, Hunan Cancer Hospital and the Affiliated Cancer Hospital of Xiangya School of Medicine, Central South University, Changsha, 410013, China; Hunan Key Laboratory of Translational Radiation Oncology, Hunan Cancer Hospital and The Affiliated Cancer Hospital of Xiangya School of Medicine, Central South University, Changsha, 410013, China; Department of Radiation Oncology, Hunan Cancer Hospital and the Affiliated Cancer Hospital of Xiangya School of Medicine, Central South University, Changsha, 410013, China; Hunan Key Laboratory of Translational Radiation Oncology, Hunan Cancer Hospital and The Affiliated Cancer Hospital of Xiangya School of Medicine, Central South University, Changsha, 410013, China; Department of Radiation Oncology, Hunan Cancer Hospital and the Affiliated Cancer Hospital of Xiangya School of Medicine, Central South University, Changsha, 410013, China; Hunan Key Laboratory of Translational Radiation Oncology, Hunan Cancer Hospital and The Affiliated Cancer Hospital of Xiangya School of Medicine, Central South University, Changsha, 410013, China; Department of Radiation Oncology, Hunan Cancer Hospital and the Affiliated Cancer Hospital of Xiangya School of Medicine, Central South University, Changsha, 410013, China; Hunan Key Laboratory of Translational Radiation Oncology, Hunan Cancer Hospital and The Affiliated Cancer Hospital of Xiangya School of Medicine, Central South University, Changsha, 410013, China; Department of Radiation Oncology, Hunan Cancer Hospital and the Affiliated Cancer Hospital of Xiangya School of Medicine, Central South University, Changsha, 410013, China; Hunan Key Laboratory of Translational Radiation Oncology, Hunan Cancer Hospital and The Affiliated Cancer Hospital of Xiangya School of Medicine, Central South University, Changsha, 410013, China; Department of Radiation Oncology, Hunan Cancer Hospital and the Affiliated Cancer Hospital of Xiangya School of Medicine, Central South University, Changsha, 410013, China; Hunan Key Laboratory of Translational Radiation Oncology, Hunan Cancer Hospital and The Affiliated Cancer Hospital of Xiangya School of Medicine, Central South University, Changsha, 410013, China; Department of Radiation Oncology, Hunan Cancer Hospital and the Affiliated Cancer Hospital of Xiangya School of Medicine, Central South University, Changsha, 410013, China; Hunan Key Laboratory of Translational Radiation Oncology, Hunan Cancer Hospital and The Affiliated Cancer Hospital of Xiangya School of Medicine, Central South University, Changsha, 410013, China; Hunan Key Laboratory of Translational Radiation Oncology, Hunan Cancer Hospital and The Affiliated Cancer Hospital of Xiangya School of Medicine, Central South University, Changsha, 410013, China; Department of Radiation Oncology, Hunan Cancer Hospital and the Affiliated Cancer Hospital of Xiangya School of Medicine, Central South University, Changsha, 410013, China; Hunan Key Laboratory of Translational Radiation Oncology, Hunan Cancer Hospital and The Affiliated Cancer Hospital of Xiangya School of Medicine, Central South University, Changsha, 410013, China; Department of Radiation Oncology, Hunan Cancer Hospital and the Affiliated Cancer Hospital of Xiangya School of Medicine, Central South University, Changsha, 410013, China; Hunan Key Laboratory of Translational Radiation Oncology, Hunan Cancer Hospital and The Affiliated Cancer Hospital of Xiangya School of Medicine, Central South University, Changsha, 410013, China; Department of Radiation Oncology, Hunan Cancer Hospital and the Affiliated Cancer Hospital of Xiangya School of Medicine, Central South University, Changsha, 410013, China; Hunan Key Laboratory of Translational Radiation Oncology, Hunan Cancer Hospital and The Affiliated Cancer Hospital of Xiangya School of Medicine, Central South University, Changsha, 410013, China

**Keywords:** nasopharyngeal carcinoma, body composition, skeletal muscle area, visceral adipose tissue density, chemo-immunotherapy

## Abstract

**Background:**

Reliable biomarkers for risk stratification and treatment monitoring in recurrent/metastatic nasopharyngeal carcinoma (R/M NPC) treated with first-line chemo-immunotherapy remain limited. This study evaluated the prognostic value of baseline computed tomography (CT)-derived body composition parameters and their early dynamic changes.

**Methods:**

In this single-center retrospective cohort study, patients with R/M NPC receiving first-line chemo-immunotherapy were included. The primary endpoint was progression-free survival (PFS), and secondary endpoints included overall survival (OS), objective response rate (ORR), and disease control rate (DCR). Associations between body composition and outcomes were analyzed.

**Results:**

A total of 199 patients were included, and 99 with post-treatment CT after 2 cycles were further analyzed for dynamic changes. Higher baseline skeletal muscle area (SMA) was independently associated with longer PFS (HR = 0.56; 95% CI, 0.37-0.85; *P *= 0.006) and OS (HR = 0.46; 95% CI, 0.23-0.93; *P *= 0.03), and with higher ORR (82.8% vs 70.0%; *P *= 0.033) and DCR (98.0% vs 91.0%; *P *= .031). During early treatment, a ≤1.3% decrease or an increase in SMA was independently associated with longer PFS (HR = 0.53; *P *= 0.045) and OS (HR = 0.27; *P *= 0.018). A greater decline in visceral adipose tissue density (**Δ**VATD < −0.9%) was also associated with more favorable PFS and OS, whereas no significant difference in ORR was observed.

**Conclusion:**

Baseline SMA and early changes in SMA and VATD have prognostic value in R/M NPC treated with first-line chemo-immunotherapy. CT-based body composition assessment may serve as a practical imaging biomarker for risk re-stratification and individualized management.

Implications for practice CT-based body composition assessment may provide a practical approach for individualized management of patients with recurrent/metastatic nasopharyngeal carcinoma receiving chemo-immunotherapy. Incorporating body composition assessment into routine CT imaging analysis may further improve risk stratification and facilitate early identification of patients with poor prognosis. For patients with low skeletal muscle reserve or significant early muscle loss during treatment, timely nutritional assessment, exercise support, and closer clinical monitoring may be considered to optimize supportive care and treatment management.

## Introduction

Nasopharyngeal carcinoma (NPC) is an epithelial malignancy with a distinct geographic distribution and a strong association with Epstein-Barr virus (EBV) infection. Although locoregional control has improved substantially in non-metastatic NPC with the application of intensity-modulated radiotherapy and modern multimodal treatment strategies, a considerable proportion of patients eventually develop recurrence and/or distant metastasis, which remains one of the major causes of NPC-related death. At this stage of disease, systemic therapy is the cornerstone of disease control. In recent years, multiple randomized phase III studies have reported that, in the first-line treatment of recurrent/metastatic NPC (R/M NPC), the addition of PD-1 inhibitors to platinum-based chemotherapy improves clinical outcomes, leading chemo-immunotherapy to gradually become a new standard treatment strategy in many regions.^1–3^ However, the identification of patients most likely to benefit from this approach, as well as the implementation of treatment monitoring and individualized treatment adjustment, remains to be further clarified.

It is well recognized that cancer development, progression, and prognosis are closely related to patients’ nutritional status. Body mass index (BMI) is a commonly used clinical indicator for nutritional assessment because it is simple and easy to apply. However, it cannot distinguish between muscle and fat. One study showed that, in individuals without cancer, BMI was only moderately correlated with directly measured body fat percentage, with correlation coefficients ranging from 0.58 to 0.75.^4^ Gonzalez et al observed that some obese-only individuals, some sarcopenic-only patients, and all those with sarcopenic obesity fell within the normal BMI range.^5^ Therefore, BMI still has important limitations as an indicator of nutritional status. In contrast, computed tomography (CT)-based body composition analysis can more accurately reflect patients’ nutritional status and assess its association with prognosis, demonstrating considerable potential for clinical nutritional assessment.

Accordingly, body composition has been regarded as a potential prognostic biomarker in multiple tumor types.^6^ In a large study investigating body composition and clinical outcomes in patients with metastatic melanoma treated with immune checkpoint inhibitors (ICIs), patients with higher muscle mass and low or moderate fat appeared to have better outcomes than those with high fat and low muscle mass.^7^ Recent studies have also demonstrated the prognostic relevance and clinical utility of body composition analysis in identifying high-risk disease at initial diagnosis in early-stage lung cancer and in predicting adverse outcomes in patients with advanced non-small cell lung cancer (NSCLC) receiving systemic therapy.^6^^,^^8^ Importantly, CT-based body composition analysis can be quantified using routine imaging performed for staging and response evaluation, making it scalable and readily implementable in clinical practice. It may therefore represent a feasible imaging biomarker platform in patients with R/M NPC receiving first-line chemo-immunotherapy. Nevertheless, evidence regarding the use of body composition analysis to predict prognosis and treatment response in patients with R/M NPC receiving first-line chemo-immunotherapy remains limited, and no clear consensus has yet been reached.

Therefore, the present study aimed to evaluate the associations of pretreatment CT-derived body composition characteristics, and dynamic changes during treatment with survival outcomes and treatment response in patients receiving first-line chemo-immunotherapy, in order to identify prognostic factors with potential clinical utility and provide new evidence for risk stratification and individualized treatment decision-making in patients with R/M NPC.

## Methods

### Study objective, design, and ethics

This study aimed to evaluate the prognostic and predictive value of contrast-enhanced CT-derived body composition parameters in patients with R/M NPC receiving first-line chemo-immunotherapy. Based on previous findings, we hypothesized that body composition and its dynamic changes are closely associated with prognosis and treatment efficacy in patients with R/M NPC. To address this question, we conducted a single-center retrospective cohort study including eligible patients treated at Hunan Cancer Hospital between June 2019 and December 2024. This study strictly adhered to the ethical principles of the Declaration of Helsinki (2013 revision) and was approved by the institutional ethics committee (Expedited Review No. 29 in 2026). The study conformed to the STROBE guidelines.

### Patient selection

The inclusion criteria were as follows: (1) age ≥ 18 years; (2) histologically confirmed non-keratinizing NPC (differentiated or undifferentiated type); (3) unresectable locally recurrent or metastatic disease with at least one measurable lesion per Response Evaluation Criteria in Solid Tumors (RECIST) v1.1; (4) receipt of at least one cycle of immune checkpoint inhibitor plus chemotherapy as first-line treatment; (5) an Eastern Cooperative Oncology Group performance status (ECOG PS) score of 0 to 2 at recurrence or metastasis; and (6) availability of traceable electronic medical records, complete imaging data for response evaluation, and survival follow-up data. The exclusion criteria were missing clinical data, absence of archived baseline contrast-enhanced CT images in our hospital, CT scan coverage not including the third lumbar vertebral level (L3), or locally recurrent disease could be treated with curative reirradiation or surgery.

### Immunotherapy and chemotherapy

All patients received immune checkpoint inhibitor-based chemo-immunotherapy. Treatment regimens included tislelizumab (200 mg on day 1),^2^ camrelizumab (200 mg on day 1),^1^ or toripalimab (240 mg on day 1),^3^ in combination with gemcitabine (1000 mg/m² on days 1 and 8) and cisplatin (80 mg/m², administered either as a single dose on day 1 or fractionated over days 1-3). This regimen was administered intravenously every 3 weeks for a total of 4 to 6 cycles. Thereafter, patients without disease progression continued anti-PD-1 monotherapy every 3 weeks until disease progression, intolerable toxicity, or refusal of further treatment.

### Body composition assessment

CT images were obtained at baseline and within 1 month after 2 cycles of chemo-immunotherapy. Images were analyzed at the third lumbar vertebral (L3) level using Slice-O-matic software (v5.0, TomoVision). This software has been widely used in body composition studies.^9–11^ Skeletal muscle and adipose tissue areas were segmented using standard Hounsfield unit (HU) thresholds.

Skeletal muscle area (SMA), subcutaneous adipose tissue (SAT), and visceral adipose tissue (VAT) were quantified. Derived indices included skeletal muscle index (SMI), skeletal muscle gauge (SMG), visceral adipose tissue index (VATI), subcutaneous adipose tissue index (SATI), total adipose tissue index (TATI), and VAT-to-SAT ratio (VSR). SMI, VATI, SATI, and TATI were normalized to height squared (m²), while SMG was calculated as skeletal muscle area × muscle density.

Early changes after 2 cycles were calculated as percentage change from baseline for SMA, VAT, SAT, skeletal muscle density (SMD), visceral adipose tissue density (VATD), and subcutaneous adipose tissue density (SATD) using the formula:


$$\frac{\mathrm{post}-\mathrm{treatment}\;\mathrm{value}-\mathrm{baseline}\;\mathrm{value}}{\left|\mathrm{baseline}\;\mathrm{value}\right|}\times 100\%.$$

Negative values indicated decrease and positive values indicated increase.

### Data collection and response evaluation

Data were collected from the electronic medical record system, including time to recurrence/metastasis, sex, age, ECOG PS, EBV DNA level, metastatic sites, baseline blood lipid profile, serum albumin levels, height, and weight. BMI was subsequently calculated. Serum albumin levels before treatment and after 2 cycles of chemo-immunotherapy were collected to evaluate their associations with survival outcomes.

Antitumor efficacy was routinely assessed using MRI of head and neck region, and CT of other body regions, every 6 weeks for the first 6 months, every 9 weeks for the next 6 months, and every 12 weeks thereafter. Response was assessed according to the RECIST v1.1 and categorized as complete response (CR), partial response (PR), stable disease (SD), or progressive disease (PD). To ensure data consistency, accuracy, and reliability, all data were independently reviewed by 2 clinicians together with each patient’s treating oncologist.

### Study endpoints

The primary endpoint was progression-free survival (PFS), defined as the time from initiation of chemo-immunotherapy to disease progression or death, whichever occurred first. Secondary endpoints included overall survival (OS), objective response rate (ORR), and disease control rate (DCR). OS was defined as the time from initiation of chemo-immunotherapy to death from any cause or last follow-up. ORR was defined as the proportion of patients whose best overall response was PR or CR. DCR was defined as the proportion of patients whose best overall response was CR, PR, or SD.

### Statistical analysis

Continuous variables were expressed as medians (interquartile range, IQR), and categorical variables as frequencies. Comparisons of baseline characteristics between groups were performed using the Student’s *t* test or Wilcoxon test for continuous variables and the chi-square test or Fisher’s exact test for unordered categorical variables. OS and PFS were estimated using the Kaplan-Meier method and compared using the log-rank test.

Continuous variables were handled in grouped analyses as follows. For baseline body composition parameters, previous studies have shown substantial sex-related differences in their distributions; therefore, sex-specific medians were used as cutoffs to dichotomize each parameter. For percentage changes in body composition after 2 treatment cycles, the overall median was used as the cutoff for dichotomization. Baseline serum albumin levels and changes in serum albumin after 2 cycles of chemo-immunotherapy were also dichotomized according to the overall median values. Cox proportional hazards models were used to evaluate associations between body composition, serum albumin levels, and outcomes. Multivariable models adjusted for age, ECOG PS, liver metastasis, and EBV DNA level; sex was additionally adjusted for change variables. Propensity score matching was performed with a caliper of 0.01. Spearman correlation analysis assessed associations between body composition parameters and lipid indices. A 2-sided *P* < .05 was considered statistically significant. Statistical analyses were performed using SPSS 26.0 and R 4.4.2.

## Results

### Patient characteristics

**Figure 1.**
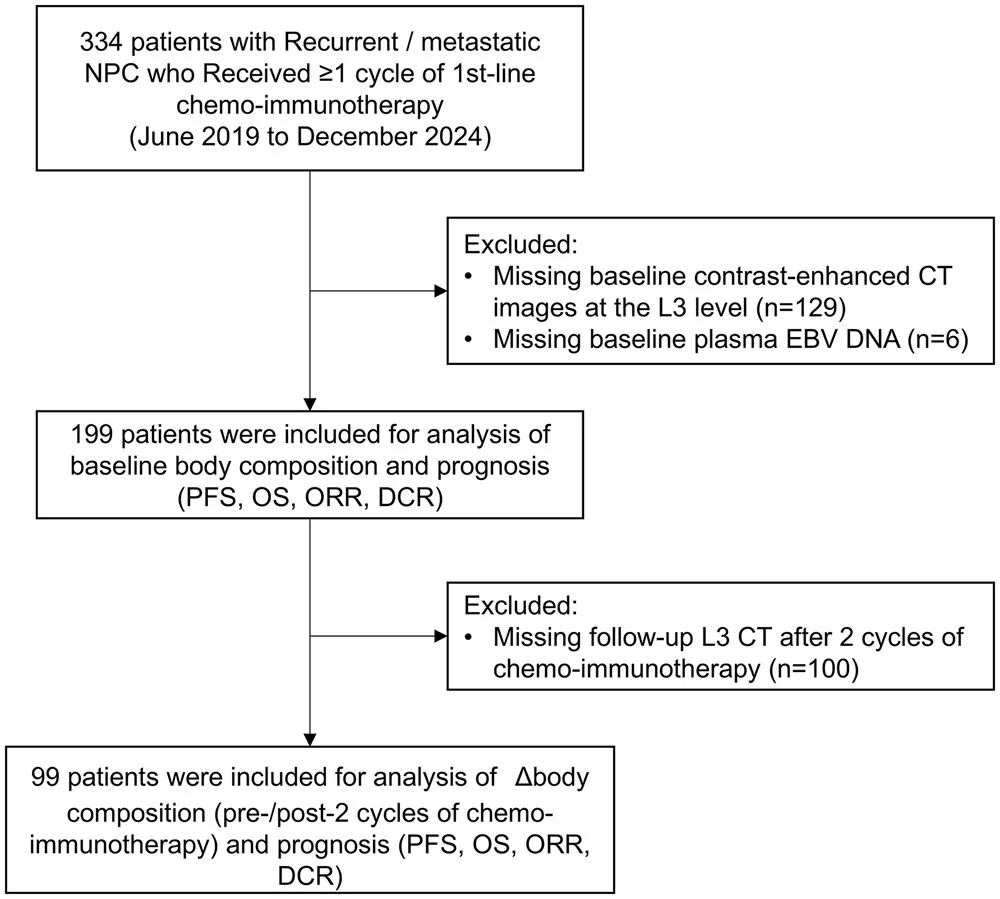
Flowchart of patient selection. Abbreviations: DCR, disease control rate; EBV, Epstein–Barr virus; ORR, objective response rate; OS, overall survival; PFS, progression-free survival; CT, computed tomography; L3, the third lumbar vertebra.

Among 334 consecutive patients with R/M NPC who received first-line chemo-immunotherapy, 129 lacked CT images suitable for body composition analysis. Among these patients, 66 (51.2%) had PET-CT examinations with unavailable CT images in our institutional Picture Archiving and Communication System, 29 (22.5%) underwent abdominal ultrasonography instead of abdominal CT for baseline evaluation, and 34 (26.4%) had baseline abdominal CT performed at external hospitals without accessible imaging data. An additional 6 patients were excluded due to missing baseline EBV DNA levels. Ultimately, 199 patients were included in the study. The patient selection flowchart is shown in Figure 1, and baseline characteristics are summarized in Table 1. The included cohort (*n* = 199) and the CT-unavailable cohort (*n* = 129) showed comparable PFS (*P *= .306, Figure S1a, see online supplementary material for a color version of this figure) and OS (*P *= .947; Figure S1b, see online supplementary material for a color version of this figure). Most baseline characteristics were balanced between groups (Table S1).

**Table 1.** Baseline characteristics of the study population (overall and matched cohorts).

Variable	Overall (*n* = 199)	Before PSM			After PSM		
		Low SMA (*n* = 100)	High SMA (*n* = 99)	*P* value	Low SMA (*n* = 82)	High SMA (*n* = 82)	*P* value
**Sex, *n* (%)**							
** Male**	162 (81.4)	81 (81.0)	81 (81.8)	.882	66 (80.5)	66 (80.5)	1.000
** Female**	37 (18.6)	19 (19.0)	18 (18.2)		16 (19.5)	16 (19.5)	
**Age, *n* (%)**							
** median [IQR], years**	50 [41-57]	52.5 [41-58]	49 [41-55]		50 [39-55]	50 [42-56]	
** <60**	176 (88.4)	85 (85.0)	91 (91.9)	.127	75 (91.5)	75 (91.5)	1.000
** ≥60**	23 (11.6)	15 (15.0)	8 (8.1)		7 (8.5)	7 (8.5)	
**ECOG PS, *n* (%)**							
** 0**	73 (36.7)	38 (38.0)	35 (35.4)	.699	31 (37.8)	34 (41.5)	.632
** 1-2**	126 (63.3)	62 (62.0)	64 (64.6)		51 (62.2)	48 (58.5)	
**Baseline plasma EBV DNA level, *n* (%)**							
** <10 000 IU/mL**	153 (76.9)	72 (72.0)	81 (81.8)	.100	61 (74.4)	64 (78.0)	.582
** ≥10 000 IU/mL**	46 (23.1)	28 (28.0)	18 (18.2)		21 (25.6)	18 (22.0)	
**Current disease stage, *n* (%)**							
** Local recurrent**	29 (14.6)	11 (11.0)	18 (18.2)	.151	10 (12.2)	14 (17.1)	.377
** Metastasis**	170 (85.4)	89 (89.0)	81 (81.8)		72 (87.8)	68 (82.9)	
**Liver metastasis, *n* (%)**							
** Yes**	65 (32.7)	36 (36.0)	29 (29.3)	.313	26 (31.7)	29 (35.4)	.620
** No**	134 (67.3)	64 (64.0)	70 (70.7)		56 (68.3)	53 (64.6)	
**Bone metastasis, *n* (%)**							
** Yes**	77 (38.7)	38 (38.0)	39 (39.4)	.840	27 (32.9)	30 (36.6)	.623
** No**	122 (61.3)	62 (62.0)	60 (60.6)		55 (67.1)	52 (63.4)	
**Lung metastasis, *n* (%)**							
** Yes**	64 (32.2)	35 (35.0)	29 (29.3)	.389	32 (39.0)	24 (29.3)	.188
** No**	135 (67.8)	65 (65.0)	70 (70.7)		50 (61.0)	58 (70.7)	
**Immune checkpoint inhibitor regimen administered, *n* (%)**							
** Camrelizumab**	90 (45.2)	45 (45.0)	45 (45.5)	.798	37 (45.1)	38 (46.3)	.746
** Tislelizumab**	56 (28.1)	30 (30.0)	26 (26.3)		23 (28.0)	19 (23.2)	
** Toripalimab**	53 (26.6)	25 (25.0)	28 (28.3)		22 (26.8)	25 (30.5)	
**ALB, *n* (%)**							
** <42.3 g/L**	100 (50.3)	49 (49.0)	51 (51.5)	.723	41 (50.0)	43 (52.4)	.755
** ≥42.3g/L**	99 (49.7)	51 (51.0)	48 (48.5)		41 (50.0)	39 (47.6)	

PSM used 1:1 nearest-neighbor matching without replacement on prespecified covariates (age, ECOG PS, baseline EBV DNA level, and presence of liver).

Abbreviations: EBV, Epstein–Barr virus; ECOG PS, Eastern Cooperative Oncology Group performance status; IQR, interquartile range; IU/mL, international units per milliliter; PSM, propensity score matching.

As of June 13, 2025, the median follow-up time was 24.0 months. At the last follow-up, 162 patients (81.4%) were alive. During follow-up, 95 patients (47.7%) experienced PFS events (disease progression or death), and 37 patients (18.6%) died (Table S1). In the overall cohort, the median PFS was 23.1 months (95% CI, 15.4-30.8 months), whereas the median OS had not yet been reached. The overall objective response rate (ORR) was 76.4%, and the disease control rate (DCR) was 94.5%. The median interval from baseline CT to initiation of immunotherapy was 9 days (IQR, 5-16 days).

The median age of the patients was 50 years (IQR, 41-57 years), and 81.4% were male. An ECOG performance status score of 1-2 was observed in 63.3% of patients. At recurrence/metastasis, 76.9% of patients had baseline EBV DNA <10 000 IU/mL, whereas 23.1% had EBV DNA ≥10 000 IU/mL. Distant metastasis was present in 85.4% of patients, including liver metastasis in 32.7%, bone metastasis in 38.7%, and lung metastasis in 32.2%. 105 (52.8%) patients did not receive radiotherapy, 60 (30.2%) patients received radiotherapy to the primary tumor, 26 (13.1%) patients received radiotherapy to metastatic lesions, and 8 (4.0%) patients received radiotherapy to both the primary tumor and metastatic lesions.

### Sex-related differences in body composition

A schematic illustration of body composition segmentation is shown in Figure S2 (see online supplementary material for a color version of this figure). Figure S2a and b (see online supplementary material for a color version of this figure) demonstrate that 2 patients with the same BMI had markedly different distributions of muscle and fat, whereas Figure S2a and c (see online supplementary material for a color version of this figure) show that, in the same patient, BMI remained unchanged before and after treatment despite a decrease in SMA and a marked increase in SAT. Significant sex-related differences in body composition distribution were observed (Figure S3, see online supplementary material for a color version of this figure). Male patients had significantly higher SMA and VAT than female patients (median SMA: 132.5 cm² vs 93.0 cm²; *P *< .001; median VAT: 73.6 cm² vs 47.1 cm²; *P *= .017), whereas SAT was higher in female patients (median SAT: 96.5 cm² vs 122.1 cm²; *P *= .001).

Based on these differences, sex-specific medians were used as cutoffs to categorize each body composition parameter into high- and low-value groups for subsequent analyses, thereby minimizing potential sex bias and improving stratification accuracy (specific cutoffs are shown in Table S2).

### Association between SMA and prognosis

**Figure 2.**
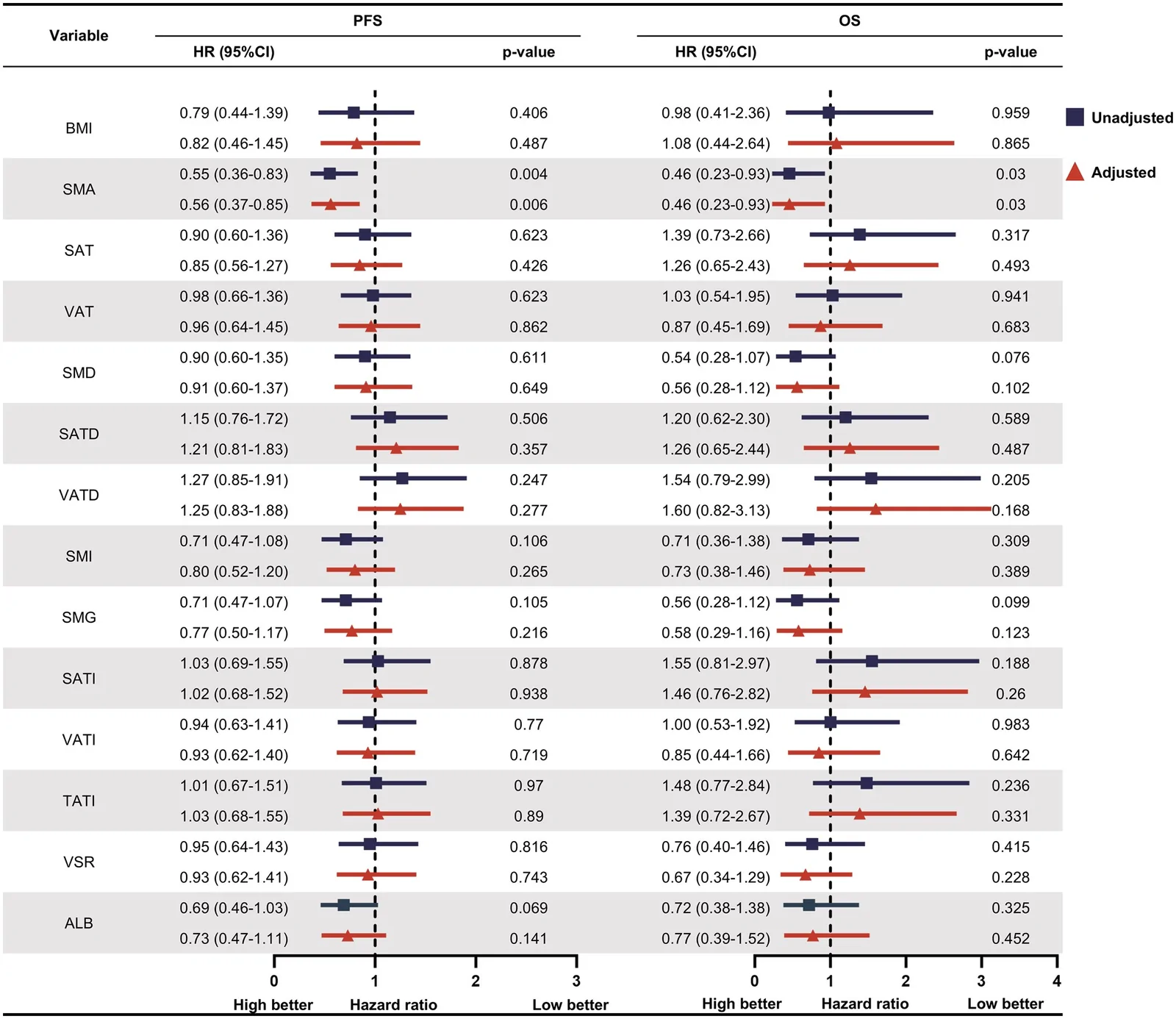
Associations of baseline serum albumin and body composition parameters with progression-free survival and overall survival. Abbreviations: BMI, body mass index; SMA, skeletal muscle area; SAT, subcutaneous adipose tissue; VAT, visceral adipose tissue; SMD, skeletal muscle density; SATD, subcutaneous adipose tissue density; VATD, visceral adipose tissue density; SMI, skeletal muscle index; SMG, skeletal muscle gauge; SATI, subcutaneous adipose tissue index; VATI, visceral adipose tissue index; TATI, total adipose tissue index; VSR, VAT-to-SAT ratio; ALB, Albumin.

Among all parameters evaluated, only SMA showed a stable and significant association with survival outcomes (Figure 2).

In univariable analysis, patients in the high-SMA group had significantly better PFS (HR = 0.55; 95% CI, 0.36-0.83; *P *= .004) and OS (HR = 0.46; 95% CI, 0.23-0.93; *P *= .03) than those in the low-SMA group. After adjustment for age, ECOG performance status, liver metastasis, and baseline EBV DNA level, high SMA remained an independent protective factor for both PFS (HR = 0.56; 95% CI, 0.37-0.85; *P *= .006) and OS (HR = 0.46; 95% CI, 0.23-0.93; *P *= .03). In a sensitivity analysis, baseline serum albumin was additionally included as a covariate in the multivariable Cox regression models. After further adjustment for serum albumin, high SMA remained independently associated with superior PFS and OS (Table S3).

**Figure 3.**
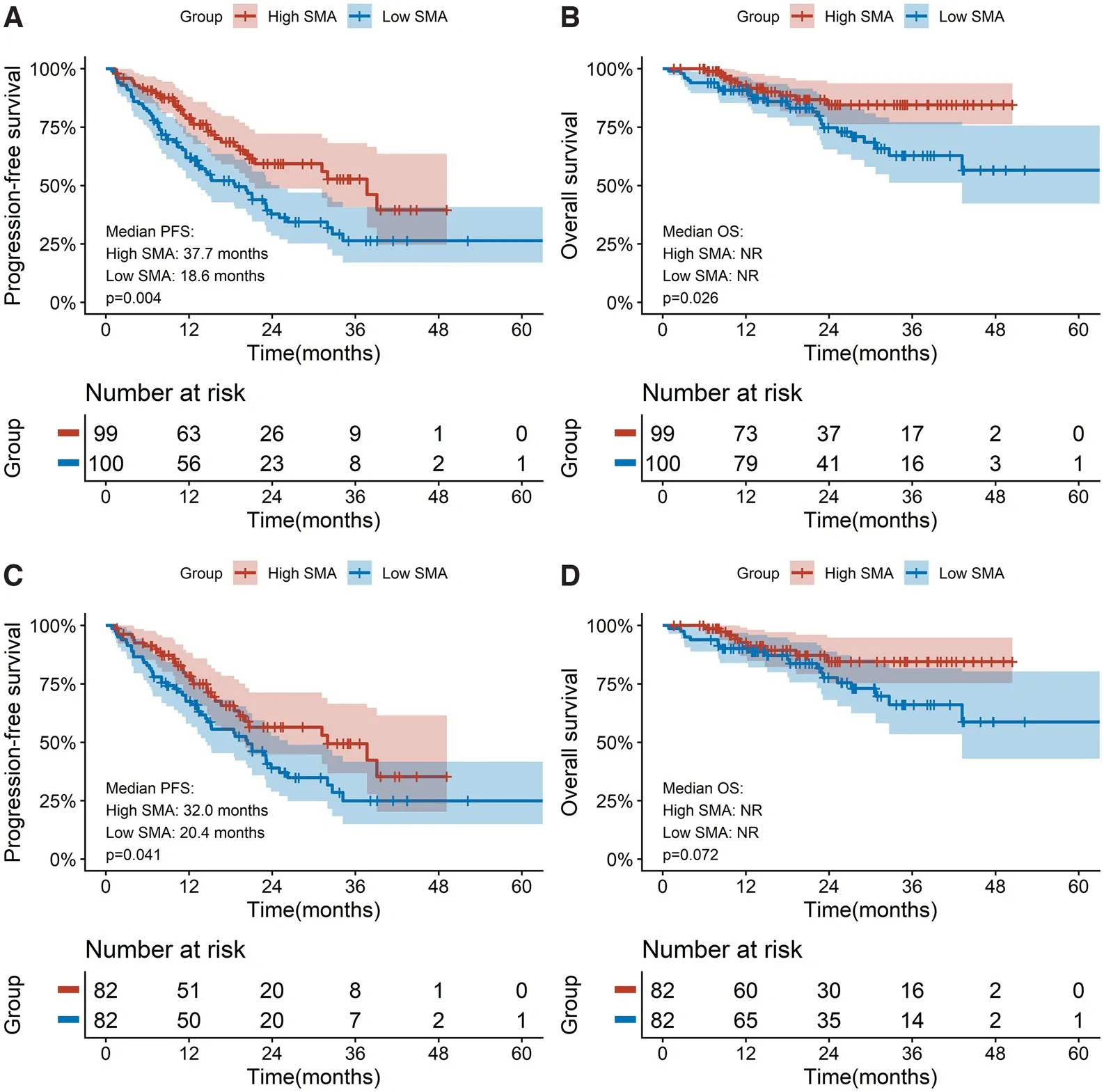
Survival outcomes by skeletal muscle area (SMA) status. Kaplan–Meier estimates of progression-free survival (PFS) and overall survival (OS) in patients with recurrent/metastatic nasopharyngeal carcinoma treated with first-line chemo-immunotherapy. (A, B) Overall cohort (n = 199), showing PFS and OS, respectively. (C, D) Propensity score-matched cohort (n = 164; 82 matched pairs), showing PFS and OS, respectively..

Kaplan-Meier analysis showed that the median PFS was 37.7 months (95% CI, 29.6-45.8 months; Figure 3A) in the high-SMA group and 18.6 months (95% CI, 12.8-24.5 months; Figure 3A) in the low-SMA group (*P *= .004). The 1- and 2-year PFS rates were 78.8% and 59.3% in the high-SMA group, compared with 62.0% and 37.9% in the low-SMA group, respectively. Median OS was not reached in either group (*P *= .026; Figure 3B). The 1- and 2-year OS rates were 94.2% and 84.6% in the high-SMA group, compared with 90.9% and 74.8% in the low-SMA group, respectively.

After 1:1 propensity score matching, 82 matched pairs were obtained, and baseline characteristics were well balanced between the 2 groups. The high-SMA group continued to show a PFS advantage (32.0 months vs 20.4 months; *P *= .041; Figure 3C), whereas no significant difference in OS was observed (*P *= .072; Figure 3D).

In addition, the high-SMA group had significantly higher ORR (82.8% vs 70%; *P *= .033; Figure S4a, see online supplementary material for a color version of this figure) and DCR (98% vs 91%; *P *= .031, Figure S4b, see online supplementary material for a color version of this figure) than the low-SMA group. No significant associations with survival outcomes were observed for the other body composition parameters, including SMD, VAT, and SAT.

### Sensitivity analysis

To address the potential confounding effect of radiotherapy, radiotherapy status was additionally included as a covariate in the multivariable Cox regression models. After adjustment for radiotherapy, baseline SMA remained independently associated with PFS (HR = 0.57; 95% CI, 0.37-0.87; *P *= .008) and OS (HR = 0.45; 95% CI, 0.22-0.92; *P *= .029) (Table S4).

Furthermore, stratified analyses were performed according to radiotherapy status. Among patients without radiotherapy, those with higher SMA showed a trend toward longer PFS (32.0 months vs 14.6 months; *P *= .085; Figure S5a, see online supplementary material for a color version of this figure), whereas OS did not differ significantly between groups (not reached vs 43.2 months; *P *= .101; Figure S5b, see online supplementary material for a color version of this figure). Among patients who received radiotherapy, higher SMA remained associated with improved PFS (not reached vs 23.1 months; *P *= .024; Figure S5c, see online supplementary material for a color version of this figure), while the difference in OS was not statistically significant (both not reached; *P *= .199; Figure S5d, see online supplementary material for a color version of this figure).

### Subgroup analysis

**Figure 4.**
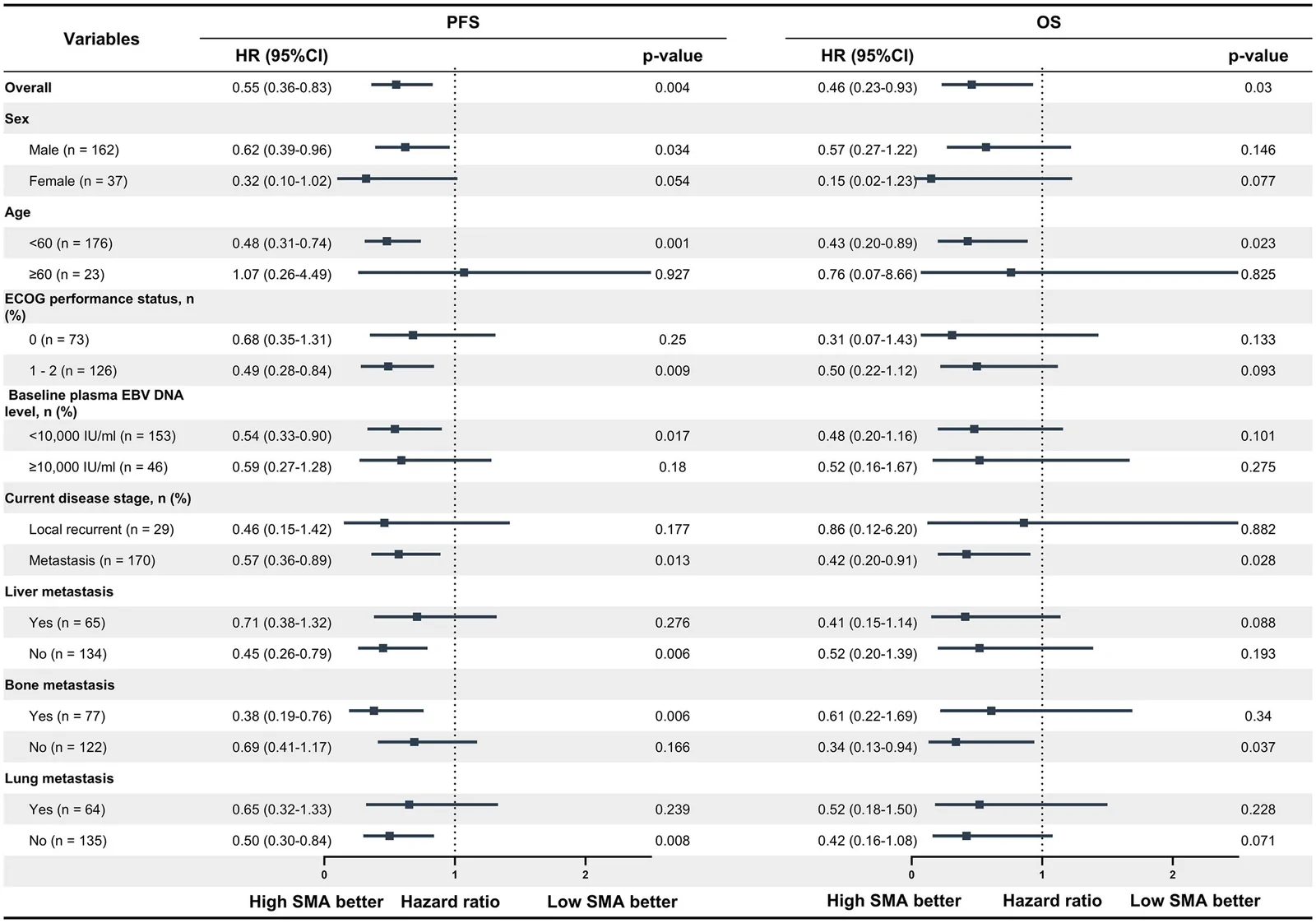
Subgroup analyses of the association between baseline skeletal muscle area and survival outcomes.

The subgroup analysis results are shown in Figure 4. Overall, the protective effects of high SMA on PFS and OS were directionally consistent across most prespecified clinical subgroups (all HRs < 1).

For PFS, high SMA reached statistical significance in the subgroups of males, age < 60 years, ECOG PS 1-2, baseline EBV DNA <10 000 IU/mL, metastatic disease, absence of liver metastasis, presence of bone metastasis, and absence of lung metastasis. A similar trend was observed in the OS analysis, with high SMA showing a protective effect across multiple subgroups.

### Association between body composition changes and prognosis

Because 100 patients lacked post-treatment CT images at the L3 level after 2 cycles of chemo-immunotherapy, 99 patients were included in the subsequent analysis (Figure 1). Their baseline characteristics are presented in Table S5. The median interval from the second cycle of immunotherapy to CT examination was 23 days (IQR, 21-26 days), and the median interval from treatment initiation to CT examination was 45 days (IQR, 42-51 days).

**Figure 5.**
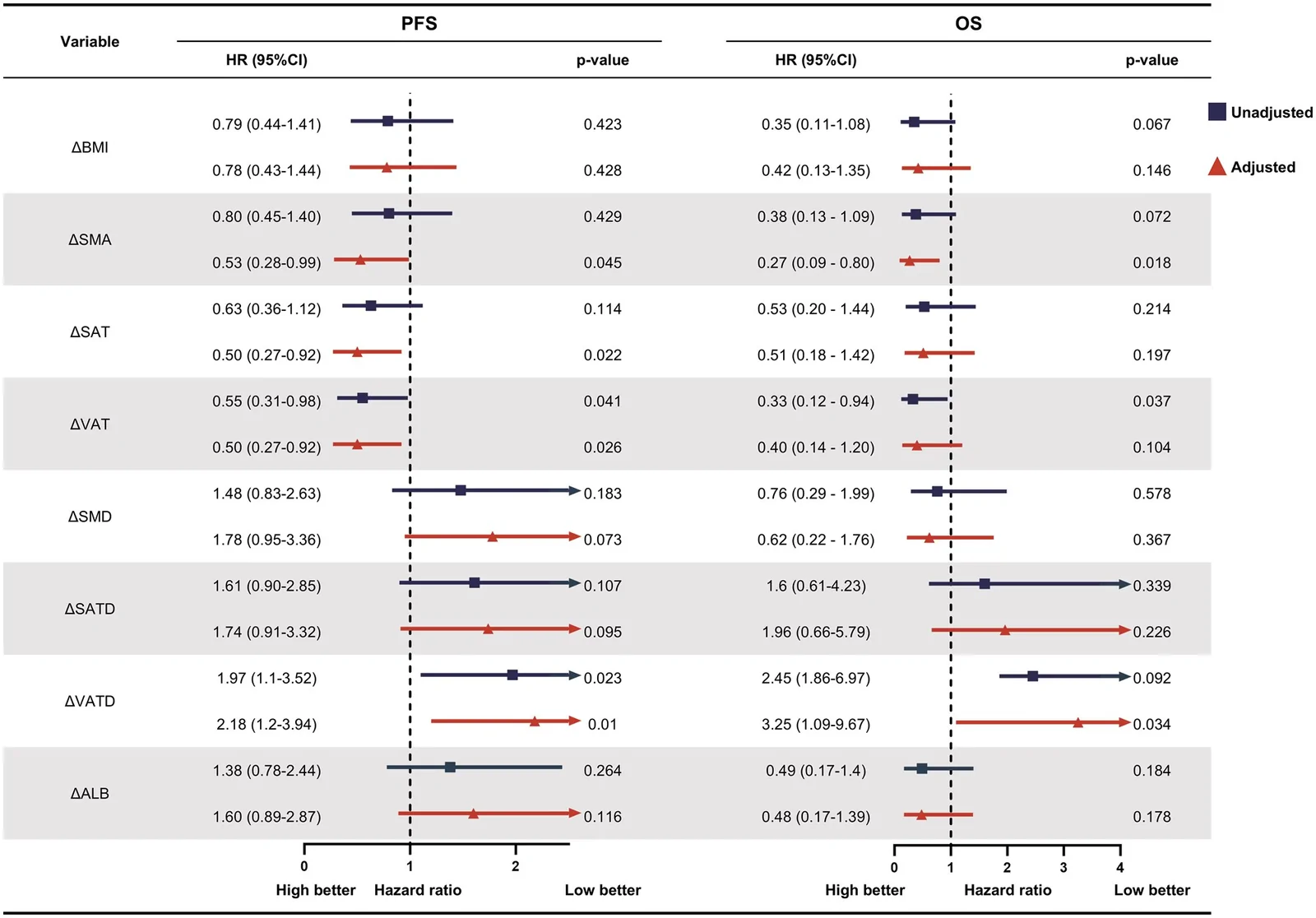
Associations of early changes in serum albumin and body composition parameters with progression-free survival and overall survival. Abbreviations: BMI, body mass index; SMA, skeletal muscle area; SAT, subcutaneous adipose tissue; VAT, visceral adipose tissue; SMD, skeletal muscle density; SATD, subcutaneous adipose tissue density; VATD, visceral adipose tissue density; ALB, Albumin.

Table S6 summarizes early changes in body composition parameters. SMA, SATD, and VATD tended to decrease, whereas SAT, VAT, and SMD tended to increase. Figure 5 shows the associations between treatment-related changes in serum albumin, body composition parameters, and prognosis. Multivariable analysis demonstrated that ΔSMA and ΔVATD were prognostic factors for both PFS and OS, independent of sex, age, ECOG performance status, liver metastasis, and baseline EBV DNA level. Compared with the ΔSMA < −1.3% group (ie, skeletal muscle area decrease > 1.3%), the ΔSMA ≥ −1.3% group (ie, skeletal muscle area decrease ≤1.3% or increase) had longer PFS (HR = 0.53; *P *= .045) and OS (HR = 0.27; *P *= .018). In contrast, compared with the ΔVATD < −0.9% group (ie, ΔVATD decrease > 0.9%), the ΔVATD ≥ −0.9% group (ie, ΔVATD decrease ≤0.9% or increase) had worse PFS (HR = 2.18; *P *= .010) and OS (HR = 3.25; *P *= .034).

In terms of treatment response, the ΔVATD < −0.9% group (ie, ΔVATD decrease >0.9%) showed a trend toward improved DCR (100% vs 90%; *P *= .056), but no significant difference in ORR was observed (Figure S6, see online supplementary material for a color version of this figure).

### Exploratory analysis

Because blood lipid testing was not a routine examination in patients with nasopharyngeal carcinoma, only 98 patients had baseline lipid profiles available. Therefore, an exploratory analysis was performed in these 98 patients to examine the associations between body composition parameters and lipid-related indices. As shown in Figure S7 (see online supplementary material for a color version of this figure), triglyceride levels were moderately positively correlated with VAT (r = 0.53; *P *< .001) and moderately negatively correlated with VATD (r = −0.513; *P *< .001). Correlations between the remaining body composition parameters and lipid-related indices were generally weak.

## Discussion

In this study, we systematically evaluated the associations of baseline body composition and its early dynamic changes with prognosis in patients with R/M NPC receiving first-line chemo-immunotherapy. We found that baseline SMA was an independent predictor of both PFS and OS. Patients with high SMA had approximately twice the mPFS of those with low SMA (37.7 months vs 18.6 months). More importantly, dynamic changes in skeletal muscle area and fat density during early treatment also had independent prognostic value: patients with a decrease in skeletal muscle area of no more than 1.3% or an increase after 2 treatment cycles, as well as those with a ΔVATD decrease >0.9%, had significantly improved PFS and OS. These findings suggest that body composition may undergo quantifiable remodeling during treatment. Collectively, our results highlight that CT-based body composition assessment derived from routine imaging not only reflects patients’ baseline physiological reserve but may also serve as a dynamic imaging biomarker to optimize prognostic stratification and individualized treatment strategies in patients receiving first-line chemo-immunotherapy. To our knowledge, this is the first study in R/M NPC to evaluate the associations of both baseline body composition and its dynamic changes with prognosis.

Our analyses showed that higher baseline SMA and less decline or an increase in SMA during early treatment were independently associated with improved PFS and OS in patients with R/M NPC. These findings are consistent with previous studies in multiple cancer types. Kim et al retrospectively analyzed 149 patients with microsatellite-stable gastric cancer treated with PD-1 inhibitors and reported that CT-defined sarcopenia was associated with poorer outcomes after ICI therapy; patients with sarcopenia had significantly shorter PFS than those without sarcopenia (1.4 months vs 2.6 months; *P *= .026).^12^ Song et al found that skeletal muscle loss during concurrent chemoradiotherapy was associated with a significantly increased risk of death in patients with small cell lung cancer.^13^ Amitani et al reported that skeletal muscle loss during neoadjuvant chemoradiotherapy predicted poor prognosis in breast cancer.^14^ Similarly, Nishioka et al investigated the association between sarcopenia and anti-PD-1 immunotherapy outcomes using the change rate of psoas major muscle area at the L2-L3 level and showed that patients with a PMMA reduction rate <10% had significantly better PFS (204 days vs 47 days; *P *= .002) and ORR (41% vs 0%; *P *= .015) than those with a reduction rate ≥10%.^15^

From a mechanistic perspective, skeletal muscle is not only a key organ for maintaining whole-body energy metabolism but also an important participant in immune regulation. First, from the perspective of energy metabolism, skeletal muscle is the largest insulin-sensitive tissue in the human body and plays a critical role in regulating postprandial glucose and lipid homeostasis.^16^ Muscle loss may lead to insulin resistance and glucose intolerance,^17^ accompanied by lipid metabolic disturbances, thereby creating a metabolic environment more favorable to tumor progression.^18^ Second, from an immunoinflammatory perspective, skeletal muscle can secrete myokines and synthesize and release glutamine to provide essential nutrients for immune cells; accordingly, muscle loss is often associated with impaired immune function.^19^ At the same time, sarcopenia is commonly accompanied by increased pro-inflammatory cytokines and decreased anti-inflammatory cytokines,^20^ and the former may act on stromal cells to remodel the tumor microenvironment, thereby restricting antitumor immunity.^21^ Finally, from the perspective of the gut microbiome, the gut-muscle axis has attracted increasing attention in recent years, and cachexia is characterized by skeletal muscle depletion.^22^ Animal experiments by Bindels et al showed that cachectic mice exhibited markedly reduced gut microbial diversity and altered microbial community structure, characterized by decreased abundance of beneficial bacteria and increased abundance of harmful bacteria.^23^ Building on this, the clinical study by Ubachs et al further confirmed that patients with cancer cachexia had reduced fecal short-chain fatty acid levels, particularly acetate, together with increased abundance of pro-inflammatory taxa such as Proteobacteria.^24^ The composition and abundance of the gut microbiota have in turn been shown to significantly influence the efficacy of immune checkpoint inhibitors.^25^ Therefore, higher SMA may represent a more stable metabolic state, more intact immune regulatory capacity, and greater physiological reserve, thereby providing a more favorable basis for sustained and effective chemo-immunotherapy.

In our study, patients with a more pronounced early decline in VATD had better PFS and OS, suggesting that the visceral adipose tissue microenvironment may undergo favorable qualitative remodeling that accompanies treatment response. Previous multicenter and tumor-cohort studies have generally suggested that higher VATD is associated with worse survival. For example, a multicenter retrospective study by Maike et al showed that higher VATD was associated with worse OS in pancreatic cancer.^26^ Nicolas et al analyzed body composition and OS in 217 patients with chemotherapy-refractory advanced colorectal cancer and found that patients with high VATD had a 140% increased risk of death; in multivariable analysis, high visceral fat density was a major predictor of poor survival.^27^ In 142 patients undergoing laparoscopic radical gastrectomy, Bian et al reported that high VAT density was significantly associated with worse overall survival (HR = 3.357; 95% CI, 1.354-8.325), indicating that high VAT density is an independent adverse prognostic factor for long-term survival in gastric cancer.^28^

From a histological perspective, qualitative changes in adipose tissue may contribute to tumor aggressiveness and progression.^29^^,^^30^ Fat with lower HU values, corresponding to lower VATD, is more consistent with a lipid-rich phenotype dominated by mature adipocytes,^31^ whereas higher VATD may reflect adipocyte atrophy, reduced intracellular lipid content, and increased extracellular matrix fibrosis.^32^ Biopsy studies in non-human primates also support that high-density VAT is associated with lower lipid content, fibrotic transformation, and abnormal adipocyte secretory function.^33^ Dysfunctional adipocytes may secrete pro-inflammatory and pro-angiogenic factors, promote macrophage infiltration, and generate a chronic inflammatory state that facilitates tumor growth.^34^^,^^35^ Therefore, in our cohort, an early decrease in VATD may indicate a shift in visceral adipose tissue from an inflammatory or fibrotic phenotype toward a more stable lipid-storage state, which is consistent with better treatment outcomes. Notably, in our exploratory analysis, triglyceride levels were moderately positively correlated with VAT area and moderately negatively correlated with VAT density, suggesting that imaging-derived visceral fat characteristics may, to some extent, reflect systemic lipid metabolic status. However, given the limited sample size, these observations require further validation in larger studies.

Previous studies have suggested that pretreatment serum albumin may have prognostic value in head and neck cancers.^36^^,^^37^ However, the prognostic significance of serum albumin in NPC remains inconclusive. Jia et al reported that lower albumin levels were associated with poorer overall survival in middle-aged patients with NPC.^38^ In contrast, Yang et al found no significant relationship between pretreatment albumin and outcomes after definitive chemoradiotherapy.^39^ In our cohort, neither baseline serum albumin levels nor early changes in albumin during treatment were significantly associated with survival outcomes. These discrepancies may be related to differences in patient populations, treatment modalities, and albumin categorization methods. Importantly, after additional adjustment for baseline albumin, SMA remained independently associated with both PFS and OS, supporting the robustness of our findings.

Integrating body composition analysis into clinical practice may have significant implications for the individualized management of patients with R/M NPC. Routine incorporation of body composition assessment could enhance risk stratification and facilitate the early identification of patients who are at higher risk of poor outcomes. First, for patients with low baseline skeletal muscle mass or substantial muscle loss during treatment, timely nutritional evaluation and intervention, and exercise support may help preserve muscle mass and potentially maximize therapeutic benefit. Second, for patients at high risk of early disease progression, more intensive monitoring, including shorter-interval imaging assessments, could be considered to detect progressive disease earlier and allow for timely treatment modification. Currently, interventions targeting cancer-related muscle loss mainly focus on nutritional optimization and exercise training. Leucine, an essential amino acid, promotes muscle protein synthesis,^40^ and studies have shown that leucine supplementation may reduce skeletal muscle loss in patients with cancer.^41^ In addition, eicosapentaenoic acid (EPA), an omega-3 fatty acid, has been reported to increase the proportion of patients who maintain or gain skeletal muscle mass.^42^ Regarding exercise interventions, a meta-analysis including 35 trials demonstrated that resistance training combined with multi-component protein supplementation could improve muscle mass and strength.^43^ Furthermore, the “gut microbiota-muscle axis” has received increasing attention in recent years, with gut microbiota potentially contributing to skeletal muscle atrophy through inflammatory regulation.^44^ However, given the observational nature of our study, whether these interventions can improve body composition and ultimately translate into survival benefits requires validation in prospective studies.

This study has several limitations. First, this was a single-center retrospective cohort study. Although multivariable adjustment was performed and propensity score matching was applied for baseline SMA, unmeasured or residual confounding may still exist. Second, a substantial proportion of patients (129/328, 39.3%) were excluded from the analysis due to the unavailability of baseline CT images for body composition assessment, primarily because initial staging evaluations were performed at outside hospitals or other imaging methods were used instead of dedicated CT as the primary screening tool. To mitigate concerns about selection bias, we compared baseline characteristics and survival outcomes between the included and excluded cohorts and found no significant differences in PFS and OS. Nevertheless, we cannot completely exclude the possibility of residual confounding, as the missing data may not be entirely random. Third, dynamic body composition analysis included only 99 patients with follow-up CT scans after 2 cycles of treatment, and the missing 100 cases may not represent random missingness, introducing the possibility of selection bias. Future larger-scale prospective multicenter studies with more comprehensive immune and metabolic analyses are needed to validate our findings. Fourth, the number of PFS events (*n* = 95, 47.7%) and OS events (*n* = 37, 18.6%) was limited, which may have reduced the statistical power of subgroup analyses and resulted in relatively wide confidence intervals in some analyses; therefore, these findings should be interpreted with caution. Fifth, body composition thresholds were dichotomized using sex-specific medians for baseline indices and the overall median for change parameters. Although this approach facilitated clinical presentation, its generalizability is limited, and multiple comparisons may have increased the risk of chance findings. Future studies should explore externally reusable thresholds or use continuous-variable/nonlinear models for more robust validation.

## Conclusion

In conclusion, in this real-world cohort of patients with R/M NPC receiving first-line chemo-immunotherapy, baseline SMA was identified as an independent prognostic marker for both PFS and OS. More importantly, early dynamic remodeling of body composition also carried prognostic information, as changes in SMA and VATD were independently associated with PFS and OS. These findings suggest that CT-based body composition assessment derived from routine imaging may serve as a scalable host-phenotype indicator and dynamic imaging biomarker for risk re-stratification and individualized management during treatment.

## Supplementary Material

oyag355_Supplementary_Data

## Data Availability

Data will be made available after the publication of this manuscript to researchers who will address a request to the corresponding authors.
