# Supplementary material for: Computed tomography-derived body composition and early changes predict outcomes of first-line chemo-immunotherapy in recurrent/metastatic nasopharyngeal carcinoma
Source: Oncologist. 2026 Sep 3;31(10):oyag355. doi: 10.1093/oncolo/oyag355 (PMC13600379; doi:10.1093/oncolo/oyag355)
Supplement: Supplementary file 1 [file oyag355_supplementary_data.zip › Revised_Supplementary_Table.docx]

**Supplementary Table**

**CT-Derived Body Composition and Early Changes Predict Outcomes of First-Line Chemo-Immunotherapy in Recurrent/Metastatic Nasopharyngeal Carcinoma**

**Supplementary Document**

[Table S1	2](#_Toc238327518)

[Table S2	4](#_Toc238327519)

[Table S3	5](#_Toc238327520)

[Table S4	6](#_Toc238327521)

[Table S5	7](#_Toc238327522)

[Table S6	9](#_Toc238327523)

| Table S1 **Baseline characteristics of the Included cohort and CT-unavailable cohort.** | | | |
| --- | --- | --- | --- |
| Variable | Included cohort (n=199) | CT-unavailable cohort (n=129) | P |
| Sex, n (%) |  |  |  |
| Male | 162 (81.4） | 111 (86.0) | 0.272 |
| Female | 37 (18.6) | 18 (14.0) |  |
| Age, n (%) |  |  |  |
| median [IQR], years | 50 [41-57] | 50 [41-57.5] |  |
| <60 | 176 (88.4) | 113 (87.6) | 0.817 |
| ≥60 | 23 (11.6) | 16 (12.4) |  |
| ECOG PS, n (%) |  |  |  |
| 0 | 73 (36.7) | 56 (43.4) | 0.223 |
| 1-2 | 126 (63.3) | 73 (56.6) |  |
| Baseline plasma EBV DNA level, n (%) |  |  |  |
| <10,000 IU/ml | 153 (76.9) | 97 (75.2) | 0.725 |
| ≥10,000 IU/ml | 46 (23.1) | 32 (24.8) |  |
| Current disease stage, n (%) |  |  |  |
| Local recurrent | 29 (14.6) | 32 (24.8) | 0.020 |
| Metastasis | 170 (85.4) | 97 (75.2) |  |
| Liver metastasis, n (%) |  |  |  |
| Yes | 65 (32.7) | 97 (75.2) | 0.128 |
| No | 134 (67.3) | 32 (24.8) |  |
| Bone metastasis, n (%) |  |  |  |
| Yes | 77 (38.7) | 81 (62.8) | 0.787 |
| No | 122 (61.3) | 48 (37.2) |  |
| Lung metastasis, n (%) |  |  |  |
| Yes | 64 (32.2) | 99 (76.7) | 0.081 |
| No | 135 (67.8) | 30 (23.3) |  |
| Immune checkpoint inhibitor regimen administered, n (%) |  |  |  |
| Camrelizumab | 90 (45.2) | 67 (51.9) | 0.493 |
| Tislelizumab | 56 (28.1) | 32 (24.8) |  |
| Toripalimab | 53 (26.6) | 30 (23.3) |  |
| PFS events | 95 (47.7) | 61 (47.3) | 1.000 |
| OS events | 37 (18.6) | 29 (22.5) | 0.391 |

Abbreviations: IQR, interquartile range; ECOG PS, Eastern Cooperative Oncology Group performance status; EBV, Epstein–Barr virus; IU/ml, international units per millilite

| Table S2 **Sex-specific median cutoffs for baseline body composition parameters** | | | | | | | | | | | | | |
| --- | --- | --- | --- | --- | --- | --- | --- | --- | --- | --- | --- | --- | --- |
|  | SMA （cm^2^） | SAT （cm^2^） | VAT（cm^2^） | SMD （HU） | SATD（HU） | VATD（HU） | SMI（cm^2^/m^2^） | SMG | SATI（cm^2^/m^2^） | VATI（cm^2^/m^2^） | TATI（cm^2^/m^2^） | VSR |  |
| Male | 132.2 | 91.5 | 73.6 | 46.2 | -96.1 | -89.6 | 48.0 | 2174.6 | 33.7 | 26.8 | 61.4 | 0.8 |  |
| Female | 93.0 | 122.1 | 47.1 | 42.8 | -99.0 | -88.5 | 37.7 | 1661.0 | 48.9 | 19.3 | 70.8 | 0.4 |  |

Abbreviations: SMA, skeletal muscle area; SAT, subcutaneous adipose tissue; VAT, visceral adipose tissue; SMD, skeletal muscle density; SATD, subcutaneous adipose tissue density; VATD, visceral adipose tissue density; SMI, skeletal muscle index; SMG, skeletal muscle gauge; SATI, subcutaneous adipose tissue index; VATI, visceral adipose tissue index; TATI, total adipose tissue index; VSR, VAT-to-SAT ratio; HU, Hounsfield units.

| Table S3 **Sensitivity analysis of the prognostic value of baseline SMA after additional adjustment for albumin.** | | | | | |
| --- | --- | --- | --- | --- | --- |
| Variable | PFS | |  | OS | |
|  | HR (95% CI) | P |  | HR (95% CI) | P |
| Age |  |  |  |  |  |
| <60 | Ref |  |  | Ref |  |
| ≥60 | 0.64 (0.31-1.31) | 0.222 |  | 0.61 (0.18-2.07) | 0.430 |
| ECOG PS |  |  |  |  |  |
| 0 | Ref |  |  | Ref |  |
| 1-2 | 0.83 (0.55-1.26) | 0.387 |  | 1.37 (0.67-2.78) | 0.397 |
| Baseline plasma EBV DNA level |  |  |  |  |  |
| <10,000 IU/ml | Ref |  |  | Ref |  |
| ≥10,000 IU/ml | 1.67 (1.07-2.63) | 0.025 |  | 1.98 (1.00-3.89) | 0.049 |
| Liver metastasis |  |  |  |  |  |
| No | Ref |  |  | Ref |  |
| Yes | 1.39 (0.90-2.14) | 0.139 |  | 1.66 (0.85-3.27) | 0.139 |
| ALB |  |  |  |  |  |
| Low | Ref |  |  | Ref |  |
| High | 0.71 (0.46-1.09) | 0.114 |  | 0.76 (0.39-1.48) | 0.414 |
| SMA |  |  |  |  |  |
| Low | Ref |  |  | Ref |  |
| High | 0.54 (0.36-0.83) | 0.005 |  | 0.45 (0.22-0.92) | 0.027 |

Abbreviations: ECOG PS, Eastern Cooperative Oncology Group performance status; EBV, Epstein–Barr virus; IU/ml, international units per millilite; ALB, albumin.

| Table S4 **Sensitivity analysis of multivariable Cox regression models incorporating radiotherapy during first-line treatment.** | | | | | |
| --- | --- | --- | --- | --- | --- |
| Variable | PFS | |  | OS | |
|  | HR (95% CI) | P |  | HR (95% CI) | P |
| Age |  |  |  |  |  |
| <60 | Ref |  |  | Ref |  |
| ≥60 | 0.69 (0.34-1.42) | 0.314 |  | 0.85 (0.20-2.19) | 0.491 |
| ECOG PS |  |  |  |  |  |
| 0 | Ref |  |  | Ref |  |
| 1-2 | 0.84 (0.56-1.28) | 0.421 |  | 1.33 (0.65-2.75) | 0.436 |
| Baseline plasma EBV DNA level |  |  |  |  |  |
| <10,000 IU/ml | Ref |  |  | Ref |  |
| ≥10,000 IU/ml | 1.95 (1.22-3.12) | 0.005 |  | 2.46 (1.22-4.95) | 0.012 |
| Liver metastasis |  |  |  |  |  |
| No | Ref |  |  | Ref |  |
| Yes | 1.31 (0.85-2.02) | 0.224 |  | 1.45 (0.73-2.87) | 0.289 |
| ALB |  |  |  |  |  |
| Low | Ref |  |  | Ref |  |
| High | 0.69 (0.45-1.06) | 0.091 |  | 0.67 (0.34-1.31) | 0.244 |
| Radiotherapy |  |  |  |  |  |
| No | Ref |  |  | Ref |  |
| Yes | 0.59 (0.38-0.91) | 0.018 |  | 0.35 (0.17-0.75) | 0.007 |
| SMA |  |  |  |  |  |
| Low | Ref |  |  | Ref |  |
| High | 0.57 (0.37-0.87) | 0.008 |  | 0.45 (0.22-0.92) | 0.029 |

Abbreviations: ECOG PS, Eastern Cooperative Oncology Group performance status; EBV, Epstein–Barr virus; IU/ml, international units per millilite; ALB, albumin.

| Table S5 **Baseline characteristics of the 99 patients included in the analysis of early changes in body composition** | | |
| --- | --- | --- |
| Variable | Baseline (n=199) | 2-Cycle Subgroup (n=99) |
| Sex, n (%) |  |  |
| Male | 162 (81.4） | 81 (81.8) |
| Female | 37 (18.6) | 18 (18.2) |
| Age, n (%) |  |  |
| median [IQR], years | 50 [41-57] | 51 [42-58] |
| <60 | 176 (88.4) | 87 (87.9) |
| ≥60 | 23 (11.6) | 12 (12.1) |
| ECOG PS, n (%) |  |  |
| 0 | 73 (36.7) | 35 (35.4) |
| 1-2 | 126 (63.3) | 64 (64.6) |
| Baseline plasma EBV DNA level, n (%) |  |  |
| <10,000 IU/ml | 153 (76.9) | 76 (76.8) |
| ≥10,000 IU/ml | 46 (23.1) | 23 (23.2) |
| Current disease stage, n (%) |  |  |
| Local recurrent | 29 (14.6) | 11 (11.1) |
| Metastasis | 170 (85.4) | 88 (88.9) |
| Liver metastasis, n (%) |  |  |
| Yes | 65 (32.7) | 33 (33.3) |
| No | 134 (67.3) | 66 (66.7) |
| Bone metastasis, n (%) |  |  |
| Yes | 77 (38.7) | 32 (32.3) |
| No | 122 (61.3) | 67 (67.7) |
| Lung metastasis, n (%) |  |  |
| Yes | 64 (32.2) | 41 (41.4) |
| No | 135 (67.8) | 58 (58.6) |
| Immune checkpoint inhibitor regimen administered, n (%) |  |  |
| Camrelizumab | 90 (45.2) | 45 (45.5) |
| Tislelizumab | 56 (28.1) | 25 (25.3) |
| Toripalimab | 53 (26.6) | 29 (29.3) |

Abbreviations: IQR, interquartile range; ECOG PS, Eastern Cooperative Oncology Group performance status; EBV, Epstein–Barr virus; IU/ml, international units per millilite.

| Table S6 **Early changes in body composition parameters after two cycles of chemo-immunotherapy** | | | |
| --- | --- | --- | --- |
| Variable | n=99, median (IQR Q1, Q3) | Group | |
|  |  | Low | High |
| ΔBMI (%) | 0 (-1.8, 2.8) | < 0 | ≥ 0 |
| ΔSMA (%) | -1.3 (-3.9, 3.8) | < -1.3 | ≥ -1.3 |
| ΔSAT (%) | 3.6 (-6.0, 22.0) | < 3.6 | ≥ 3.6 |
| ΔVAT (%) | 3.4 (-12.6, 33.7) | < 3.4 | ≥ 3.4 |
| ΔSMD (%) | -2.6 (-10.0, 4.7) | < -2.6 | ≥ -2.6 |
| ΔSATD (%) | -0.4 (-5.8, 4.0) | < -0.4 | ≥ -0.4 |
| ΔVATD (%) | -0.9 (-5.3, 4.5) | < -0.9 | ≥ -0.9 |
| ΔALB (%) | 1.0 (-7.1, 8.7) | <1.0 | ≥ 1.0 |

Abbreviations: BMI, body mass index; SMA, skeletal muscle area; SAT, subcutaneous adipose tissue; VAT, visceral adipose tissue; SMD, skeletal muscle density; SATD, subcutaneous adipose tissue density; VATD, visceral adipose tissue density; ALB, Albumin.
