# Supplementary material for: Computed tomography-derived body composition and early changes predict outcomes of first-line chemo-immunotherapy in recurrent/metastatic nasopharyngeal carcinoma
Source: Oncologist. 2026 Sep 3;31(10):oyag355. doi: 10.1093/oncolo/oyag355 (PMC13600379; doi:10.1093/oncolo/oyag355)
Supplement: Supplementary file 1 [file oyag355_supplementary_data.zip › Supplement_Data.zip!/Revised Supplementary Figure.docx]

**Supplementary Figure**

**CT-Derived Body Composition and Early Changes Predict Outcomes of First-Line Chemo-Immunotherapy in Recurrent/Metastatic Nasopharyngeal Carcinoma**

**Supplementary Figure**

[Figure S1	2](#_Toc238327814)

[Figure S2.	3](#_Toc238327815)

[Figure S3.	4](#_Toc238327816)

[Figure S4.	5](#_Toc238327817)

[Figure S5	6](#_Toc238327818)

[Figure S6.	7](#_Toc238327819)

[Figure S7.	8](#_Toc238327820)

Figure S1**.** Survival outcomes of patients included in the included cohort (n=199) and the CT-unavailable cohort (n=129).

Figure S2. Representative CT images of body composition analysis. (a) and (b) show two patients with the same BMI but different body composition distributions. (a) and (b) show the same patient before treatment and after two cycles of chemo-immunotherapy, respectively, demonstrating that body composition changed despite an unchanged BMI.

Figure S3. Sex-related differences in baseline body composition parameters. ns, not significant (*p* ≥ 0.05); **p* < 0.05, ***p* < 0.01, ****p* < 0.001.

Figure S4. Association between baseline skeletal muscle area (SMA) and treatment response.

Figure S5. Association between baseline skeletal muscle area and survival outcomes after adjustment for radiotherapy and stratification by radiotherapy status. (a, b) Progression-free survival and overall survival in patients without radiotherapy (n=105). (c, d) Progression-free survival and overall survival in patients with radiotherapy (n=94).

Figure S6. Association between early changes in skeletal muscle area (ΔSMA) and visceral adipose tissue density (ΔVATD) and treatment response after two cycles of chemo-immunotherapy.

Figure S7. Correlations between body composition parameters and lipid-related indices (n = 98). Spearman correlation analysis was performed. Correlation strength was classified based on |r| values: 0.2–0.4 as weak, 0.4–0.6 as moderate, and 0.6–0.8 as strong.
